# Iatrogenic Right-Sided Diaphragmatic Hernia After Colonoscopy

**DOI:** 10.7759/cureus.59761

**Published:** 2024-05-06

**Authors:** Jiddu A Guart, Trenton Taros, Robert Guber, Camille Briskin

**Affiliations:** 1 Surgery, University of Massachusetts, Worcester, USA; 2 General Surgery, University of Massachusetts, Worcester, USA

**Keywords:** public health and safety, diagnostic laparoscopy, colonoscopy, diaphragmatic hernia, endoscopy

## Abstract

Congenital diaphragmatic hernias (CDH) occur as a result of genetic and environmental factors that occur during the early stages of fetal development. Overall, CDH are considered to be quite rare and are often discovered when patients are neonates. The patient in this case underwent a routine colonoscopy for high-risk polyps but then developed the sudden onset of cramping abdominal pain and PO (per os) intolerance. She was found to have a right-sided diaphragmatic hernia which ultimately required operative intervention. Retrospectively, a close review of prior imaging revealed a potential diaphragm defect. Post-colonoscopy diaphragmatic hernias are very rare and right-sided ones are rarer, making this case report an important addition to the literature.

## Introduction

Diaphragmatic hernias (DHs) occur when a diaphragmatic defect allows abdominal organs to herniate into the chest cavity. They can be divided into two main subsets: congenital diaphragmatic hernias (CDH) and acquired diaphragmatic hernias (ADH), which typically occur as a result of blunt or penetrating trauma [1].

Post-colonoscopy DH is an extremely rare adverse event and, to the best of our knowledge, only 10 cases have been reported in the literature [2-10]. Although there are no reported fatal cases, the morbidity can potentially be quite severe for colonoscopy-induced DH, with complications that can include tension pneumothorax as well as the inability to reduce scope from the thoracic cavity. DH can result in bowel strangulation and death; therefore, it is important to highlight the signs and symptoms of post-colonoscopy DH to better guide efficient diagnosis and management. In this case, we report a patient who experienced a DH after a colonoscopy, most likely due to a previously undetected congenital diaphragmatic defect.

## Case presentation

A 74-year-old female with a past medical history of actinic keratosis, anxiety, depression, gastroesophageal reflux disease, and hypothyroidism, presented to the emergency department complaining of persistent abdominal pain after colonoscopy. The patient stated that she underwent a routine colonoscopy for high-risk polyps (combination of tubular adenoma and hyperplastic polyp), at which time she had 17 polyps removed with biopsy forceps, the majority having been removed via cold snare with one being removed via hot snare. The same day as her colonoscopy, she developed cramping abdominal pain, intolerance to oral intake of both liquids and solids, and obstipation.

**Figure 1:**
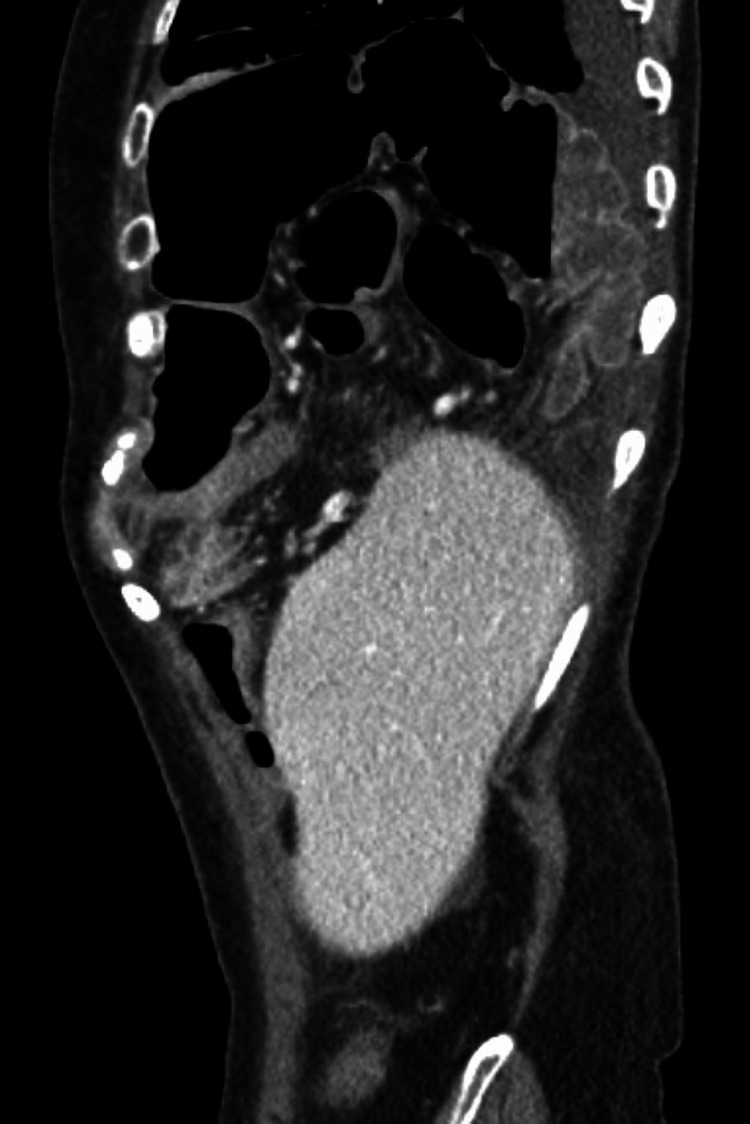
CT at presentation showing a right-sided, large diaphragmatic hernia containing portions of the ascending and transverse colon without evidence of obstruction, bowel wall thickening, or pneumatosis. Although the CT does not allow for the full examination of the right lung, it confirms total displacement and near-total collapse of the right lower lobe.

**Figure 2:**
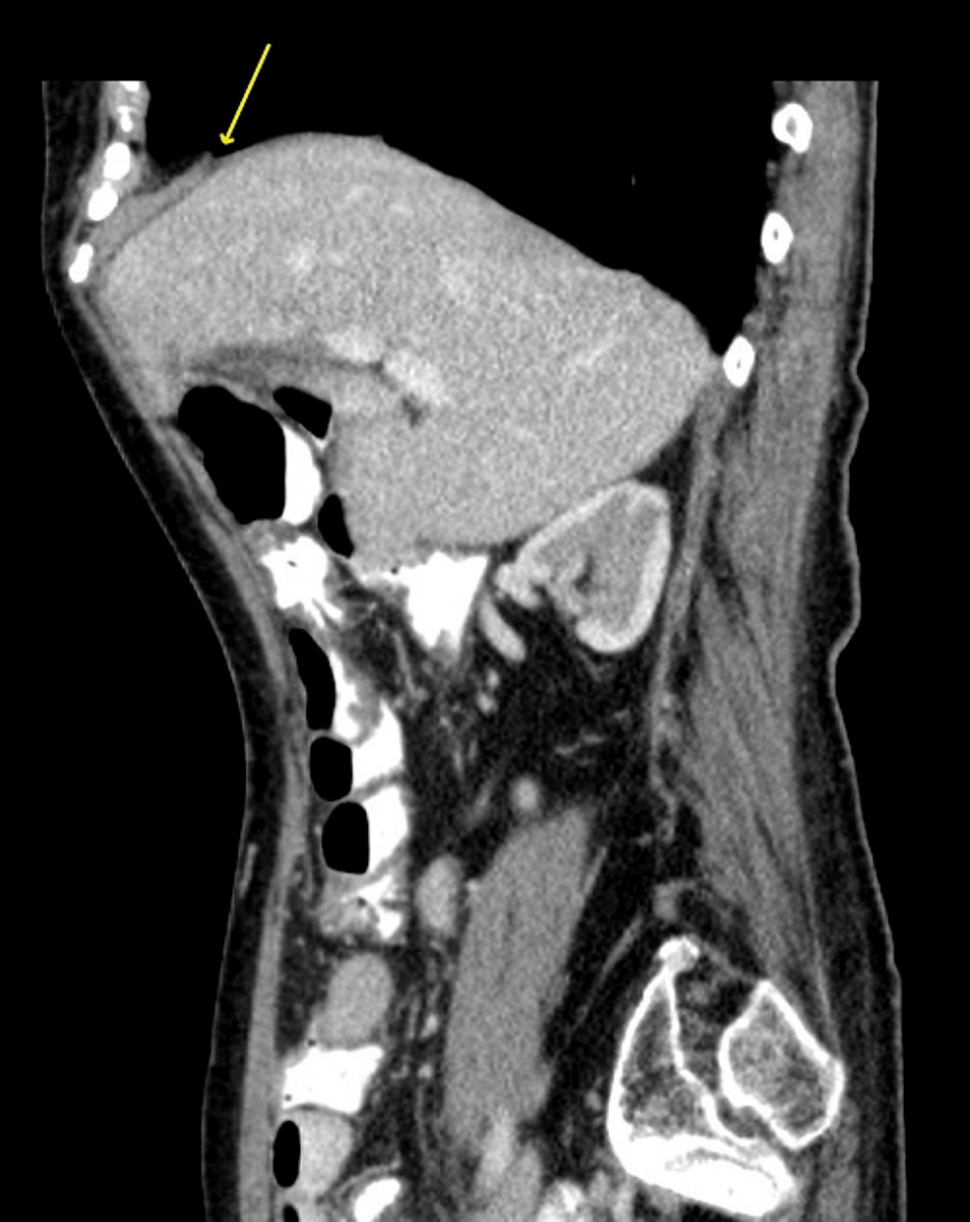
CT abdomen and pelvis from two years prior to presentation showing what appears to be a small diaphragmatic defect just anterior to the liver dome (see arrow).

On arrival at the emergency department, she was found to be afebrile and hemodynamically stable with a benign abdominal exam. Her lab work was unremarkable, but her CT scan was significant for the interval development of a moderate to large right-sided DH (as compared to her prior CT scan in the medical record), containing both right and transverse colon (Figure 1). It was at this time that general surgery was consulted. Interestingly, on a review of imaging from prior admissions, a small potential defect was noted (Figure 2).

Operative intervention

The decision was made to take the patient to the operating room for the reduction and repair of the hernia that same night urgently. The patient was placed in a classical foregut position with the legs in stirrups with the operating surgeon between the patient's legs. The abdomen was entered via a combination of the Veress needle at Palmer’s point and the Optiview entry. Three additional trocars were placed in standard foregut position with an upsizing of the left upper quadrant entry trocar. The DH was visualized in the right posterolateral position, findings consistent with a Bochdalek hernia. The hernia contents, which included the colon, were carefully reduced into the abdominal cavity. All portions of the colon appeared healthy. A 12 French red rubber catheter was placed into the defect to equalize the intrathoracic and intraabdominal pressures.

Suture was used to primarily close the diaphragmatic defect in a running fashion, and the red rubber catheter was subsequently removed. A 4 x 6 mesh was placed and secured via an endoscopic hernia stapler, ensuring 3 cm of overlap all around. The center of the mesh was secured to the peritoneal lining of the diaphragm with fasteners. The 12 mm port was closed using a fascial closure device using 0-Vicryl (Ethicon, Inc., Raritan, New Jersey, United States), and the skin was closed with 4-0 Monocryl (Ethicon, Inc.) and covered with Dermabond (Ethicon, Inc.).

The patient’s postoperative course was complicated by a clinically insignificant right-sided capnothorax treated with immediate postoperative continuous positive airway pressure (CPAP) resulting in improvement in subsequent x-rays. She recovered well but was subsequently discharged home on postoperative day 7, secondary to disposition issues. 

## Discussion

CDHs are a rare but serious medical condition that occurs during fetal development. An abnormal opening in the diaphragm allows abdominal organs to herniate into the chest cavity. This can jeopardize in-utero lung development, often resulting in life-threatening respiratory and circulatory complications after birth. The exact cause of CDH is not always clear but may be due to a combination of genetic and environmental factors during the early stages of fetal development [11]. CDH may remain undetected for extended periods or even throughout a person's life and they are often incidentally discovered during medical imaging for unrelated reasons. There are different types of CDH, each characterized by the location and size of the diaphragmatic defect.

Types of CDH

Bochdalek Hernia

This is the most common type of CDH, accounting for approximately 70-75% of cases [11]. Bochdalek hernias occur when there is a defect in the posterolateral aspect of the diaphragm. The herniation usually occurs on the left side but can also be bilateral or right-sided. Bochdalek hernias are typically diagnosed prenatally through ultrasound, and they are often associated with severe respiratory distress and lung hypoplasia at birth.

Morgagni Hernia

Morgagni hernias are less common, representing about 20-25% of all CDH cases [11]. They occur when there is a defect in the anterior portion of the diaphragm, allowing the herniation of abdominal contents into the anterior mediastinum. Morgagni hernias are usually smaller than Bochdalek hernias and are more often found on the right side. 

Central (Hiatal) Hernia

Central or Hiatal hernias are the rarest forms of CDH, making up approximately 2-5% of cases [11]. In this type of hernia, the defect occurs in the central portion of the diaphragm near the esophageal hiatus. 

Incidence and causes

The incidence of CDH varies depending on the type and geographical region. Overall, the estimated incidence of CDH is approximately 3/10,000 live births [12]. The literature estimates very few instances of iatrogenic DHs as a result of colonoscopy as only 10 cases have been reported between 1999 and the present day. Over 15 million colonoscopies are done annually in the United States reducing the widespread risk of death resulting from colon cancer by 60%. While colonoscopies are generally safe, there are risks associated, some of which require operative intervention. These are most commonly perforation, bleeding, infection, and general risks of receiving anesthesia [13].

Regarding DH from an iatrogenic standpoint, these can occur due to various factors related to the mechanics of the colonoscopy or patient anatomy. Some of these factors include tortuosity and redundancy of the colon and the presence of congenital diaphragmatic defects. During a colonoscopy, if excessive force is applied or if the colonoscope encounters resistance due to tight angles or adhesions, there is a potential for unintended pressure to be exerted on the diaphragm. This pressure, combined with any pre-existing weaknesses or defects in the diaphragm's structure, can lead to the formation of a hernia as seen in this case.

Recommendations

To mitigate the risk of iatrogenic DH following colonoscopy, healthcare professionals need to exercise caution during the procedure. Recommendations include paying attention to the amount of force applied and the angles at which the colonoscope is maneuvered. Additionally, proper patient positioning and support are crucial to prevent the development of hernias, which ultimately require operative intervention as illustrated in this case. Finally, access to fluoroscopy to identify DH intra-procedurally is important both to identify the presence of the hernia itself as well as any complications that may arise because of it.

## Conclusions

This case highlights the rare but potentially serious complication of a DH following a routine colonoscopy. Despite the infrequency of such occurrences, healthcare providers must remain vigilant, especially in patients with pre-existing risk factors such as congenital diaphragmatic defects. The prompt recognition of symptoms such as abdominal pain and PO (per os) intolerance after colonoscopy is crucial for timely diagnosis and intervention. Furthermore, a thorough review of imaging studies, even those obtained prior to the onset of symptoms, can provide valuable insights into potential predisposing factors. Surgical intervention, as demonstrated in this case, remains the mainstay of treatment for symptomatic DH, emphasizing the importance of effective collaboration between surgical and medical teams. Lastly, measures to minimize the risk of iatrogenic DH during colonoscopy, including careful technique and patient positioning, are essential to enhance patient safety and prevent such complications in the future.
